# On intersectionality: visualizing the invisibility of Black women

**DOI:** 10.1186/s41235-022-00450-1

**Published:** 2022-11-26

**Authors:** Shelby Billups, Barbara Thelamour, Paul Thibodeau, Frank H. Durgin

**Affiliations:** 1 Department of Psychology, Swarthmore College, 500 College Avenue, Swarthmore, PA 19081 USA; 2 Department of Psychology, Oberlin College, Oberlin, OH, USA; 3 Present Address: The Character Lab, Philadelphia, PA USA

**Keywords:** Intersectionality, Semantic differential, Non-prototypicality

## Abstract

Intersectionality refers to the simultaneous and interacting effects of multiple group categorization on individuals with minoritized status, often leading to being perceived in a manner inconsistent with the additive contributions of those categories. For Black women, a number of findings have contributed to the idea that Black women have a unique perceived absence of status, for example, and are perceived as distinct from being Black or a woman. We sought to quantify and visualize the combined effects of race and gender on judgments of persons using data-defined dimensions (the Semantic Differential; Osgood et al. in The measurement of meaning, University of Illinois Press, Champaign, 1957). Our data suggest that gender and race contribute to orthogonal dimensions of difference in the perception of persons. Whereas white males, white females, and Black males all seem to be perceived in accord with additive effects in these two dimensions, Black females seem to be perceived more neutrally, as if neither their gender nor their race is treated as predictive.

**Supplementary Information:**

The online version contains supplementary material available at 10.1186/s41235-022-00450-1.

## Significance statement

Racism and sexism appear to be endemic to our society, and measuring the cognitive effects of race and gender bias is of critical importance. This paper seeks to provide a data-driven framework for identifying the conceptual dimensions that are most relevant to understanding the interactions between race and gender bias, known as intersectionality. In this study, we used quantitative cognitive tools to extract principal dimensions of social judgments from a variety of rating scales. We found that two classic cognitive dimensions (good–bad and strong–weak) emerged to provide an insightful conceptual space for visualizing the separate and combined effects of race bias and gender bias on human cognition. The data in this paper can serve as a kind of model of how data-driven cognitive science can provide insight into the conceptual spaces used by people making judgments of other people. It also provides analyses of how participants with different combinations of race and gender identities perceive the judgmental biases present in society. By clarifying cognitive dimensions along which race and gender biases emerge, we aim to diversify the approaches to investigating and addressing biases in society.

## Introduction

The goal of this paper is to use cognitive methods, specifically the data-defined semantic differential (Osgood et al., 1957), to provide a visualization of how the intersections of social categories affect the perception of social value with regard to persons. Intersectionality (Crenshaw, 1989, 1991) refers to the idea that social evaluations of social categories, such as race, sexual orientation, and gender, do not seem to combine additively. Much of the empirical work used to support this idea has been focused on the special status of Black women (e.g., Levin et al., 2002). Here, we use a method for mapping semantic spaces to map out effects of gender and race separately and in combination.

Intersectionality describes the significance of belonging to multiple social categories for the individual, the ways they are perceived, and their experiences in the world (Bowleg, 2012; Cole, 2009). This framework, deriving from Black feminist studies, has been used to examine prejudice and discrimination that is experienced within the context of membership in multiple disadvantaged social groups (e.g., race, gender, sexual orientation, class). Both with regard to anticipated gender or race discrimination, and with respect to rates of sex-based and raced-based harassment, Black women, who can experience both racism and sexism, seem to anticipate and experience the additive effects of both forms of discrimination, which is sometimes described as double jeopardy (Berdahl & Moore, 2006; Buchanan et al., 2009; Chaney et al., 2021, Remedios & Snyder, 2018). In contrast, with respect to prejudicial stereotypes, investigations within the intersectionality framework have observed interacting effects unique to that intersection (Donovan, 2011; Ghavami & Peplau, 2013). Embodying two disadvantaged social categories (The Combahee River Collective, 1977/2014; Crenshaw, 1991) renders Black women invisible as they represent the prototypical example of neither their race (i.e., Black men) nor their gender (i.e., white women) (Purdie-Vaughns & Eibach, 2008; Sesko & Biernat, 2018).

Experimental research on intersectionality focusing on perceptions of Black women’s invisibility has relied on several methods, including timed categorization tasks, which showed participants took longer to categorize Black women as Black and as women (Thomas et al., 2014). This dual non-prototypicality has been shown across development, as both children (Lei et al., 2020) and adults (Johnson et al., 2012) take longer to categorize Black women as feminine as a function of their racial category. Sesko and Biernat (2018) used poorer recognition memory for faces of Black women to operationalize Black female invisibility and examined its relationship to perceived prototypicality. Among their findings was that rating Black women as less warm than women in general was related to poorer recognition of Black women’s faces. Overall, these studies suggest that being both Black and a woman is related to being perceived as not sharing prototypical characteristics with either group considered separately.

The present study contributes to the existing literature by using the semantic differential to examine intersectional invisibility. The semantic differential was originally developed as a data-defined measure of connotative meaning (Osgood, 1952), and it has been adapted to a wide range of cases (Dalton et al., 2008). By looking for principal sources of variance across a variety of rating scales, it consistently provides a three-dimensional space including the orthogonal dimensions of evaluation (good–bad), potency (powerful–weak), and activity (active–passive). Williams and Morland (1976) used semantic differential scaling extensively to study the interpretation of color and race in young children, concluding that both the race, Black, and the mere color, black, are perceived as relatively negative but relatively powerful, even by young children. More recently, Kervyn et al. (2013) related the two-dimensional (warmth, competence) stereotype content model of social status (Fiske et al., 2002) to semantic differential theory, suggesting, in part, that the two-dimensional semantic spaces of their theory may approximate a 45° rotation of the two-dimensional space defined by the evaluative and potency dimensions of the semantic differential (see Cuddy et al., 2008). Their methods for studying status—having participants rate a variety of social groups (e.g., rich people, homeless people, professionals)—formed the methodological basis for our manipulation of race and gender. In particular, we followed their practice of manipulating social role within participants, but added a between-participant manipulation of gender and/or race information. We expected that our manipulations of race and gender would facilitate tests for the presence or absence of the additivity of race and gender effects in semantic differential space and thus of the unique invisibility of Black women. Moreover, they allow us to compare the designation of Black woman with the absence of any race or gender specification.

## Method

In order to measure effects of race and gender categories, we adapted a method developed for studying effects of social status (Fiske et al., 2002) and combined it with the data-driven methods of the semantic differential. Specifically, we had participants rate various social roles along a number of scales. The social roles were given with or without concomitant indications of race and/or gender. Specifically, 18 social roles (see the supplementary materials in Additional file 1) were presented either with or without race and/or gender indicators. Many of the roles were derived from the work of Cuddy et al. (2008). Roles that were considered closely related (e.g., opposites) like single and married, liberal and conservative, were kept in separate lists. Participants rated how each of the resulting social categories was regarded by society along 10 different dimensions. Rating dimensions were intended to represent 2 each of: evaluative dimensions (*virtuous*, *good*), potency dimensions (*powerful*, *resilient*), activity dimensions (*energetic*, *emotional*), warmth dimensions (*warm*, *sociable*), and competence dimensions (*competent*, *confident*).

To reduce the burden on individual raters, each participant saw only 9 of the social roles. The assignment of race and gender labels was manipulated between participants, meaning that all 9 social categories rated by a single participant included the same race and gender information. This was intended to minimize experimental demand by eliminating explicit comparison by race or gender within raters, while focusing their attention on differences between social roles. The ratings were collected online from November 6, 2019, through February 14, 2020.

### Participants and design

The preregistered design called for 1440 participants in the USA with equal subsets (360) identifying as each combination of (Black or White) race and (male or female) gender. There were two different lists of social groups, which appeared in each of 2 orders, and each was administered equally often with each of 9 distinct combinations of race and gender information, for a total of 36 unique surveys. Each unique survey was administered successfully to 40 participants with 10 participants identifying as each combination of Black or White and male or female. We excluded (and replaced) participants who differently identified their gender or race from the gender or race they had previously given the recruiting service (TurkPrime; Litman et al., 2017).

### Attention screening

In addition to requiring 90% performance on previous HITs, to ensure participant attention, a simple odd vs even number task that lasted about a minute preceded the survey. Both the attention test and the survey were administered using PsyToolkit (Stoet, 2010, 2017). The survey data of participants who got more than 20% wrong on the number task were excluded from analysis. In addition to this attention test, we had also preregistered an exclusion criterion for any participant who gave the same rating to 30 consecutive items out of the 90 tested. All excluded participants were replaced.

### Analysis

The 10 average ratings for each of the 162 combinations of race, gender, and social role were computed and submitted to principal components analysis (PCA; Dunteman, 1989) with normalized variables (scaled, centered) and singular variable decomposition (Mardia et al., 1980) with orthogonal rotation. The first three PCs accounted for 72%, 15%, and 7% of the variance in the ratings, respectively. As expected, the first PC loaded on the positive end of each rating scale and was lowest for the most negative social group, “homeless”; the second PC loaded most positively on the scale for “powerful” and was highest for the social group “rich.” These two dimensions correspond well with the *evaluative* dimension (good vs. bad) and the *potency* dimension (strong vs. weak) consistently found by semantic differential methods. The third PC, which we will not consider further, loaded highly on “energetic” and “emotional,” consistent with an *activity* dimension (active vs. passive), and the social groups “young” and “old” were at the extremes. The loadings for PC1 and PC2 were used to generate evaluative and potency values for each item for each participant based on their ratings. Note that because the potency dimension is extracted second, it is better thought of as potency in the absence of valence. More information concerning the results of the PCA is presented in supplementary materials (Additional file 1).

To estimate the effects of gender and race alone on each of the two primary dimensions, two linear mixed effects regressions (LMER) were conducted on the data using social roles as random effects with respect to the main manipulations of race or gender (but not both) and participants as random effects, with the baseline condition being the absence of any race or gender information. To estimate the effects of combinations of race and gender, a second pair of LMER analyses was conducted comparing the pairing of gender and race to the baseline condition in which neither gender nor race was specified.

## Results

**Fig. 1:**
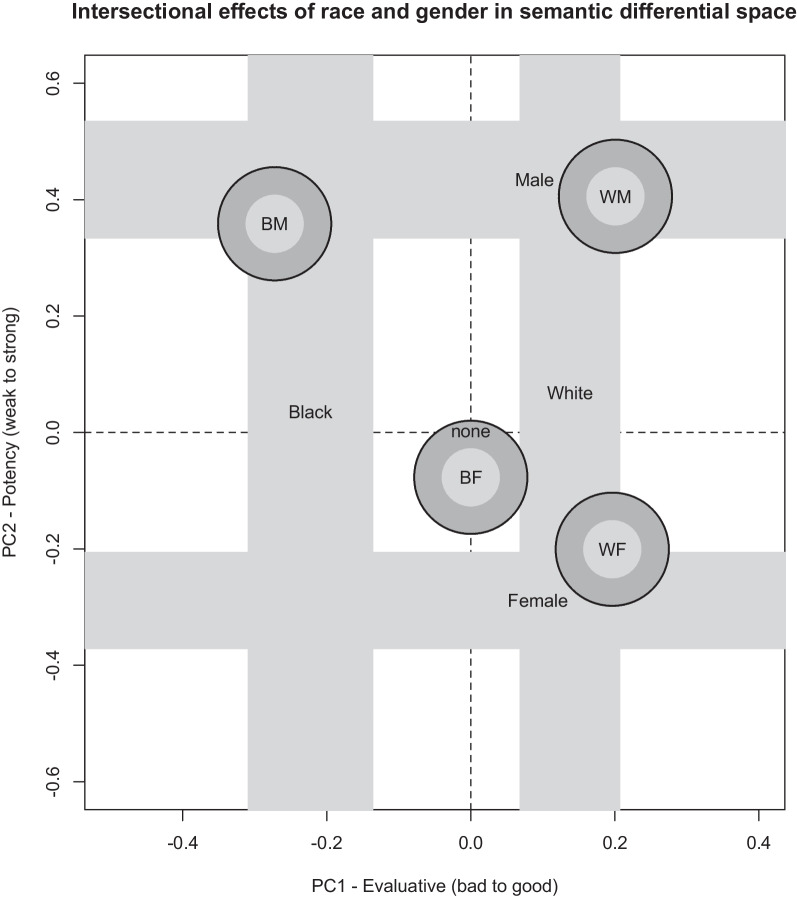
Intersectional effects of race and gender in the space traditionally defined by the semantic differential. Effects of race or gender labels alone were evident in distinct primary dimensions (evaluative and potency, respectively). Thickness of bars for each of these effects represents two standard errors of the means derived from linear mixed effects regression models comparing each single label to the baseline condition (“none”: no mention of race or gender). Circular symbols (outer radii closely approximate one standard error in each dimension) are shown for cases where both gender and race were specified. Locations of verbal labels show two-dimensional position in the semantic space with respect to the baseline condition

Figure 1 depicts the overall results by plotting the deviations from baseline that occurred as a result of specifying race, gender, or both.

### Specifying race or gender

When only one or the other was specified, race and gender seemed to have orthogonal effects. On one hand, for the evaluative dimension, the LMER indicated that specifying race as Black produced a reliable negative shift in evaluative scores, *β* =  − 0.22, *t*(49.0) = 2.58, *p* = 0.013, whereas specifying race as White produced a marginally positive shift in evaluative scores, *β* = 0.14, *t*(128.8) = 0.051, relative to leaving race unspecified. There was no reliable effect of gender designations on the evaluative dimension. On the other hand, for the potency dimension, specifying race had no reliable effect, but specifying gender as Female produced a reliable reduction in perceived potency, *β* =  − 0.29, *t*(22.6) = 3.08, *p* = 0.005, and specifying gender as Male produced a reliable increase in perceived potency, *β* = 0.43, *t*(21.2) = 4.28, *p* < 0.001.

Because race only affected the evaluative dimension and gender only affected the potency dimension, these effects are depicted in Fig. 1 as wide lines forming a box in the two-dimensional semantic space of evaluation and potency. The widths of these vertical and horizontal lines correspond to twice the magnitude of the standard errors computed in the LMER. The words “Male,” “Female,” “Black,” and “White” are presented at their respective mean locations in the two-dimensional space relative to the origin represented by the baseline condition.

### Specifying race and gender

If the effects of race and gender were additive, we should expect that combinations of race and gender would fall near to the corners of the semantic space formed by the separate effects of race and gender alone. Indeed, for three of the four combinations tested (Black Male, White Male, and White Female), LMERs found the predicted deviations both in evaluative scores, *β* =  − 0.27, *t*(51.6) = 2.99, *p* = 0.004, *β* = 0.20, *t*(119.1) = 3.08, *p* = 0.007, *β* = 0.20, *t*(103.7) = 2.61, *p* = 0.010, respectively, and in potency scores, *β* = 0.36, *t*(20.2) = 2.79, *p* = 0.011, *β* = 0.41, *t*(24.9) = 4.50, *p* < 0.001, *β* =  − 0.20, *t*(26.0) = 2.36, *p* = 0.026, respectively. These effects can be seen by the locations of the circular symbols in Fig. 1.

However, there is a fourth combination which clearly does not fit the additive model. As is evident in Fig. 1, specifying Black Female had no reliable effect on either the evaluative dimension or the potency dimension compared to the baseline condition where neither race nor gender was specified. There was no shift in the evaluative dimension, *β* = 0.00, *t*(65.7) = 0.00, *p* = 0.999, nor was there a reliable shift in the potency dimension, *β* =  − 0.08, *t*(24.4) = 0.83, *p* = 0.415.

### Participant identity (exploratory analysis)

Because the ratings were framed in terms of “how does society view…” (rather than “how do you view…”), it seems worth exploring how the race and gender identity of each of the participants related to their perception of how society judges people according to race and gender (McCormick-Huhn et al., 2019). Including participant race and gender in the LMER analyses supported the following conclusions.

When only race or gender was included in the social role categories, an LMER on the evaluative dimension showed that Black categories had lower evaluative scores relative to the case without race or gender, *β* =  − 0.30, *t*(246.0) = 2.17, *p* = 0.031, and there were no reliable interactions with participant race or gender.

When only race or gender was included in the social categories, an LMER on the potency dimension showed that female categories had lower potency scores relative to the case without race or gender, *β* =  − 0.25, *t*(60.4) = 2.11, *p* = 0.039, and male categories had elevated potency scores, *β* = 0.56, *t*(50.4) = 4.44, *p* < 0.001. However, the elevation of male categories on potency was smaller among male participants than among female participants, *β* =  − 0.26, *t*(775.3) = 2.06, *p* = 0.039. This effect of participant gender on the perception of male power is consistent with the idea that female participants are more sensitive to perceived power differences between men and women.

When both race and gender were included in social categories, an LMER on the evaluative dimension showed that both White Male categories and White Female categories had elevated evaluative scores relative to the baseline judgments for social roles without race or gender, WM: *β* = 0.30, *t*(535.5) = 2.33, *p* = 0.020, WF: *β* = 0.33, *t*(489.9) = 2.52, *p* = 0.012. Black Male categories had lowered evaluative scores overall, *β* = -0.73, *t*(239.1) = 5.20, *p* < 0.001, but this effect was modulated both by participant race, with White participants giving higher evaluative scores than Black participants, *β* = 0.57, *t*(774.9) = 3.21, *p* = 0.001, and by participant gender, with male participants giving higher estimates than female participants, *β* = 0.55, *t*(774.9) = 3.15, *p* = 0.002. The participant race effect on the evaluative scores for Black Male categories is consistent with the idea that Black participants are more aware of societally-embedded negative evaluations of Black men than are White participants.

When both race and gender were included in the social role identity, an LMER on the potency dimension showed that both White Male social categories and Black Male social categories had elevated potency scores relative to social roles without race or gender, WM: *β* = 0.48, *t*(75.1) = 4.03, *p* < 0.001, BM: *β* = 0.65, *t*(38.2) = 4.30, *p* < 0.001. For Black Male categories, however, this effect was modulated both by participant race, with Black participants giving higher potency scores for Black Males than did White participants, *β* = 0.30, *t*(775.5) = 2.35, *p* = 0.019, and by participant gender, with female participants giving higher potency scores to Black Male identities than did male participants, *β* = 0.40, *t*(775.5) = 3.07, *p* = 0.002. This latter effect is consistent with females’ overall higher ratings of potency for males, but was only reliable for Black Male social categories in the present analysis.

**Fig. 2:**
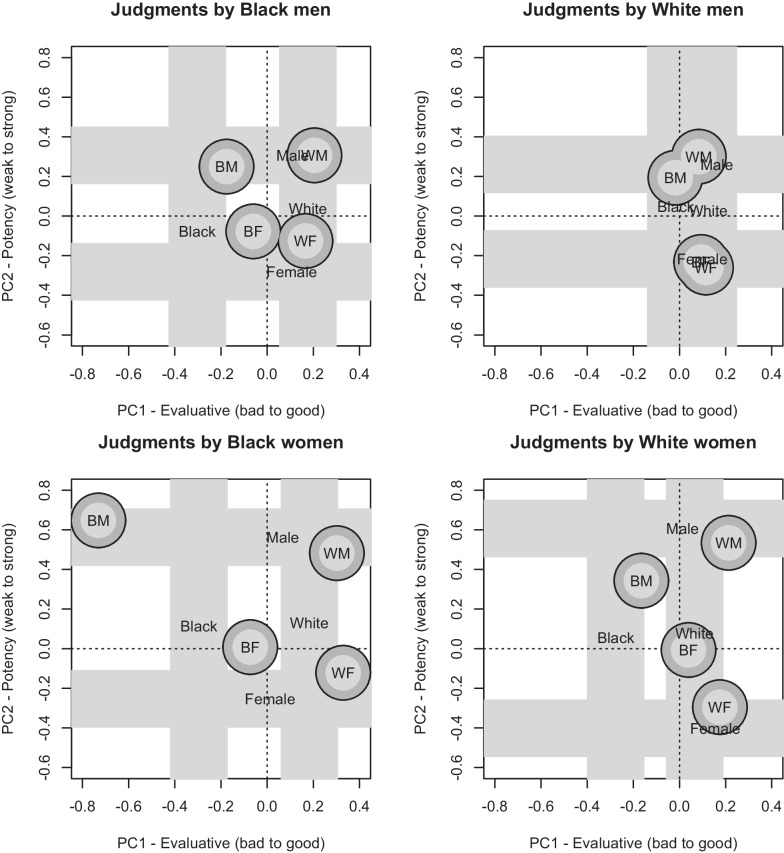
Results as a function of gender and race identity of the participant. Note that line widths and symbol sizes only approximately represent variability (two times the standard errors of the means). The zero point in each plot represents the mean scores for the cases where neither race nor gender was specified

Figure 2 shows how the main graph would look for each subset of participants. Overall, when the race and gender of participants were factored in, the main conclusions remained similar regarding effects of race, gender, and their combination. The exploratory analysis supports the idea that Black participants and Female participants were more sensitive to societal biases regarding both race-based and gender-based differences in perceived evaluation and perceived power. Notably, with respect to the issue of intersectional identities, in no analysis did the perception of Black Female social categories reliably differ from social categories where neither gender nor race was specified.

## Discussion

*Intersectionality* posits that different combinations of identity (such as with respect to race and gender) may interact in their effects on how a person is perceived. Our goal was to use the sematic differential to provide a visualization of how some combinations of race and gender identity (i.e., Black woman) depart from additive effects of these two identities alone. We used an implicit (between-subject) manipulation of race and gender in a task that focused participants on making judgments about different social roles, while race and gender were held constant for each rater. It is important to emphasize, given how our findings seem to capture perceived race and gender effects, that no participant was ever asked to rate social identities having different genders or races from each other. Using data-derived semantic dimensions, we found that our between-subject manipulations of gender and race seemed to produce two distinct semantic effects. Differences in race alone were associated exclusively with the evaluative dimension. Differences in gender alone were associated exclusively with the potency dimension.

For three of the four groupings that combined gender and race (White male, Black male, and White female) the ratings of the combination were consistent with additive contributions of race and gender information presented alone, but for Black female identities, the ratings did not differ from the case where neither race nor gender was specified. This pattern of results appears as a striking illustration of the combination of Black race and female gender as having the effect of essentially erasing both race and gender identity, thus rendering Black females invisible with respect to social stereotypes concerning women and Black people.

In our study, we observed some differences (e.g., less negative societal perceptions for Black identities among White Male participants) in the answer to our question about society’s views that are consistent with the idea that White and male individuals either paid less attention to gender and race than did other groups or believe it has less influence on societal judgments than did other groups tested. Because we did not ask individual participants to rate different racial categories, it is possible that the presence of race and gender information may have been more salient to those who experience the negative consequences of societal bias, but there could also simply be differences in perceptions of societal biases.

In this study, we used data-defined dimensions of judgment to examine a specific example of intersectionality: Black female invisibility. The identities of the participants sampled matched the social categories under investigation. Our observations could be framed in terms of an interaction between race and gender categories, leaving Black women differentially vulnerable to stereotypes and stigma about their race and gender than prototypical members of these groups. This finding lends support for examining intersectional invisibility as a unique reality of social perception on individuals with multiple marginalized categories (i.e., Donovan, 2011). Although there may be limits to how easily this technique might transfer to other combinations of identities, we believe the semantic differential model may help to provide a cognitive foothold for the study of intersectionality more generally. Abele et al. (2021) review alternative theoretically-motivated frameworks, but, as shown in the supplementary materials, the invisibility of Black women is also evident when only the warmth/competence dimensions of our ratings were considered. We think the semantic differential method has promise because it is based on orthogonal dimensions of human connotative judgment that have been shown to emerge spontaneously across many forms of human judgment and which seem to align with race and gender bias in the USA.

## Supplementary Information

**Additional file 1.** Supplementary materials.

## Data Availability

Data may be accessed at: https://osf.io/s5pz8/?view_only=1a76ed95779d4b74a277968327ec7cf4. The preregistration of the study is available at: https://aspredicted.org/yg2q3.pdf.
