# Supplementary material for: On intersectionality: visualizing the invisibility of Black women
Source: Cogn Res Princ Implic. 2022 Nov 26;7:100. doi: 10.1186/s41235-022-00450-1 (PMC9701302; doi:10.1186/s41235-022-00450-1)
Supplement: Supplementary file 1 — Additional file 1. Supplementary materials. [file 41235_2022_450_MOESM1_ESM.docx]

**Supplementary Materials**

**1. Details of the Principal Components Analysis (PCA)**

Tables S1 and S2 provide the loadings and variability accounted for by the various PCs. Consistent with prior studies, PC1 corresponds to the Evaluative (negative-positive) dimension and PC2 corresponds to the Potency (weak-strong) dimension.

Table S1. The PCA loadings on the 10 rating scales:

PC1 PC2 PC3 PC4 PC5 PC6 PC7 PC8 PC9 PC10

Sociable 0.347 -0.035 0.189 -0.575 0.197 -0.527 0.199 -0.204 -0.091 -0.325

Confident 0.344 0.261 0.150 0.105 0.286 -0.021 0.436 -0.039 0.146 0.696

Virtuous 0.343 -0.168 -0.305 0.095 0.105 0.558 0.381 -0.330 -0.319 -0.272

Energetic 0.306 0.211 0.489 -0.373 -0.463 0.485 -0.163 0.073 0.044 0.023

Powerful 0.323 0.357 0.033 0.257 0.422 0.093 -0.129 0.450 0.282 -0.464

Good 0.326 -0.293 -0.340 -0.087 -0.063 0.016 -0.301 -0.308 0.698 0.085

Emotional 0.123 -0.604 0.641 0.416 0.140 -0.020 -0.086 -0.087 -0.004 -0.044

Competent 0.354 0.188 -0.112 0.132 0.119 -0.130 -0.656 -0.205 -0.511 0.212

Warm 0.284 -0.481 -0.249 -0.216 -0.036 -0.013 0.012 0.702 -0.196 0.214

Resilient 0.343 0.116 -0.087 0.447 -0.661 -0.386 0.232 0.042 -0.014 -0.141

Table S2. Importance of components:

PC1 PC2 PC3 PC4 PC5 PC6 PC7 PC8 PC9 PC10

Standard deviation 2.68 1.22 0.86 0.46 0.40 0.28 0.22 0.18 0.14 0.12

Proportion of variance 0.720 0.150 0.074 0.022 0.016 0.008 0.005 0.003 0.002 0.001

Cumulative proportion 0.720 0.870 0.943 0.965 0.981 0.989 0.993 0.997 0.999 1.000

Note that the dimensions that emerged did not differ when PCAs were conducted separately for participants with different race and gender identities. Moreover, we tested this formally using confirmatory factor analysis, and found that no evidence that the model structure differed depending on the race and/or gender of the participants.

Although the analyses in the paper were based on PCs computed on item-wise averages, we replicated the main analyses using PCs generated across the raw data set at the suggestion of a reviewer. The statistical results were essentially identical: The evaluative dimension differed by race alone, but not gender, Black, β = -0.53, *t*(49.1) = 2.57, *p* = .014; White, β = 0.32, *t*(128.7) = 1.93, *p* = .056. The Potency dimension differed by gender alone, but not race, Female, β = -0.31, *t*(22.8) = 3.14, *p* = .005; Male, β = 0.49, *t*(21.0) = 4.41, *p* < .001. The Evaluative dimension differed only for Black men, β = -0.67, *t*(49.9) = 3.03, *p* = .004, White women, β = 0.48, *t*(103.7) = 2.68, *p* = .009, and White men, β = 0.45, *t*(122.6) = 2.57, *p* = .011. The Potency dimension differed only for Black men, β = 0.39, *t*(20.3) = 2.86, *p* = .010, White women, β = -0.21, *t*(26.4) = 2.30, *p* = .029, and White men, β = 0.47, *t*(24.6) = 4.73, *p* < .001.

**2. Plot of Social Roles in Evaluative/Potency space**

The eighteen social roles used in the study are plotted in their average locations in the Evaluative/Potency space in Figure S1. Note that few of the social roles were negative, and that none of the negative roles were rated as powerful. It is expected that roles such as “criminal” and “felon” would have fallen into that part of the space, had they been used.

*Figure S1*. A plot of the average evaluative and potency scores for each of the 18 social roles used in the study.

**3. Naming the social groups.**

In the baseline condition we used the locutions “people”, “parents”, “Christians”, “political activists”, “educated people”, “unemployed people”, “immigrants”, “Americans”, “young people”, “old people”, “married people”, “single people”, “liberals”, “conservatives”, “working class people”, “professionals”, “homeless people”, and “rich people”. When combining these with gender and/or race, we consulted Google for the most common orderings of these adjectives or nouns with gender and race information. For example, typically adjectival social roles came first, with race as adjective and gender as noun: “rich men” or “rich black people”, “professional black women”, “conservative black women”, and nouns sometimes came at the end, “black male political activists”, and could even incorporate gender: “white mothers”, but some words used as nouns at baseline became adjectives when combined with gender information: “white immigrant women” (but “white immigrants”), “black Christian women”, and “white American men”.

**4. Plotting the Effects of Race and Gender in Warmth/Competence space**

Four of our rating scales can be used to construct Warmth (Warm and Sociable scales) and Competence (Competent and Confident) scales consistent with the stereotype content model (Cuddy et al., 2008; Fiske et al., 2002). The scaled ratings on these two constructs were fairly highly correlated in our data (*r* = 0.67). A plot of the effects of race and/or gender within these dimensions is shown in Figure S2.

*Figure S2*. Effects of race and gender plotted in Warmth/Competence space

A striking feature of Figure S2 is that, relative to the case where no gender or race is given, gender effects for male and female appear to fall, separately, along each of the two dimensions, with females (and white females) seen as warmer and males (and white males) seen as more competent than ungendered social categories (and ungendered white social categories). However, this means that the category white does not fall in between white male and white female categories, as it did in the semantic differential space. The special *invisible* status of Black women (identical to the absence of race and gender information) is replicated in this space. However, this space suggests little differentiation between describing someone simply as Black, and describing them as Black and male, whereas these two categories were clearly distinguished by the Potency dimension of the semantic differential, β = 0.32, *t*(43.3) = 5.023, *p* < .001. Only in the upper right sector does there appear to be rough additivity of the vectors for gender and the vector for white to produce white male and white female locations.

Thus, although the main diagonal axis of Figure S2 is roughly consistent with the Evaluative dimension of the semantic differential space, the potency dimension (assuming a rotation by 45°) appears to be somewhat compressed in the upper right sector and fully collapsed in the lower left sector.

**5. Aligning Warmth/Competence Space with Evaluative/Potency Space**

Figure S3 shows a 45° rotation of a plot of social roles in Warmth/Competence space, (with the Warmth axis plotted with negative values upward). The 45° rotation of Warmth/Competence space reproduces a vertically compressed version of Figure S1, with most of the discriminating information carried by the horizontal (evaluative) axis. Because Warmth and Competence are both positive traits, they are correlated, and there is little evidence of low-warmth, high competence individuals. Only two categories: old (warm but incompetent) and conservative (competent but cold) are far removed from the quadrants where warmth and competence are either both present or both absent. Again, the figure suggests that some of the information carried by the potency axis in Figure S1 (e.g., the difference between young and old) is missing from warmth/competence space.


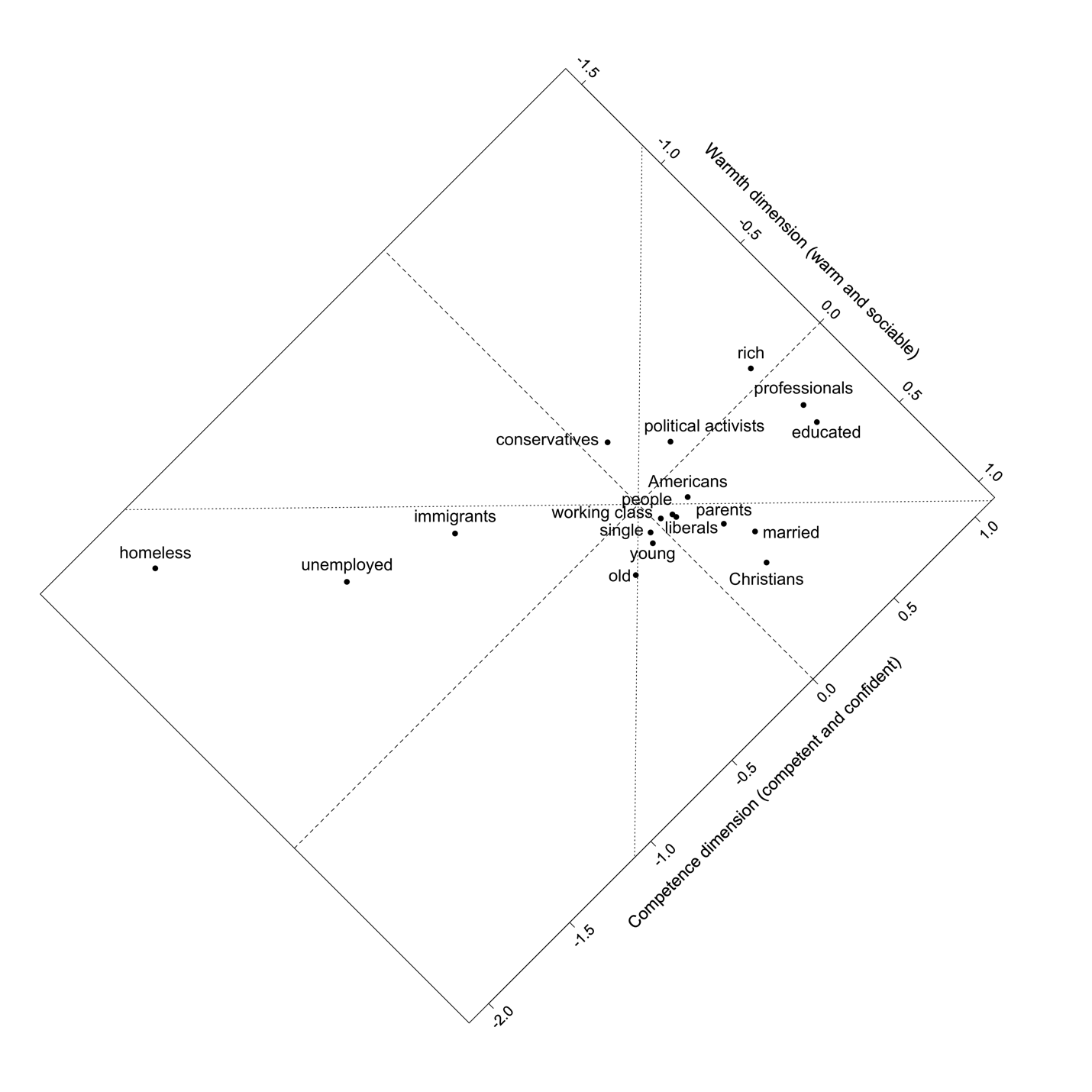


*Figure S3*. A 45° rotated image of a plot of the scaled average Warmth (warm, sociable) and Competence (competent, confident) scores for each of the 18 social roles used in the study. Note that the Warmth axis is oriented with negative values “up”, so that the plot rotation conforms to the Semantic Differential space shown in Figure S1. In the rotated image, the horizontal line appears to represent the evaluative axis, and the vertical line the potency axis. From the origin, positive competence is good and strong; positive warmth is good and weak.
